# Do Cost Functions for Tracking Error Generalize across Tasks with Different Noise Levels?

**DOI:** 10.1371/journal.pone.0136251

**Published:** 2015-08-27

**Authors:** Jonathon Sensinger, Adrian Aleman-Zapata, Kevin Englehart

**Affiliations:** 1 Institute of Biomedical Engineering, University of New Brunswick, Fredericton, New Brunswick, Canada; 2 Department of Electrical and Computer Engineering, University of New Brunswick, Fredericton, New Brunswick, Canada; 3 Department of Electronics and Computer Engineering, University of Guadalajara, Guadalajara, Jalisco, Mexico; Queen's University Belfast, UNITED KINGDOM

## Abstract

Control of human-machine interfaces are well modeled by computational control models, which take into account the behavioral decisions people make in estimating task dynamics and state for a given control law. This control law is optimized according to a cost function, which for the sake of mathematical tractability is typically represented as a series of quadratic terms. Recent studies have found that people actually use cost functions for reaching tasks that are slightly different than a quadratic function, but it is unclear which of several cost functions best explain human behavior and if these cost functions generalize across tasks of similar nature but different scale. In this study, we used an inverse-decision-theory technique to reconstruct the cost function from empirical data collected on 24 able-bodied subjects controlling a myoelectric interface. Compared with previous studies, this experimental paradigm involved a different control source (myoelectric control, which has inherently large multiplicative noise), a different control interface (control signal was mapped to cursor velocity), and a different task (the tracking position dynamically moved on the screen throughout each trial). Several cost functions, including a linear-quadratic; an inverted Gaussian, and a power function, accurately described the behavior of subjects throughout this experiment better than a quadratic cost function or other explored candidate cost functions (p<0.05). Importantly, despite the differences in the experimental paradigm and a substantially larger scale of error, we found only one candidate cost function whose parameter was consistent with the previous studies: a power function (cost ∝ error^α^) with a parameter value of α = 1.69 (1.53–1.78 interquartile range). This result suggests that a power-function is a representative function of user’s error cost over a range of noise amplitudes for pointing and tracking tasks.

## Introduction

People implicitly make control decisions using estimates of task dynamics and the various costs associated with that task [1]. This framework may be modeled using optimal control methods for the various stages of control, such as state / parameter estimation and control regulation [2]. The behavior predicted by these models closely matches that of humans in a variety of situations [3], and even where it doesn’t, the framework provides insight into why humans don’t behave optimally (e.g., [4]). The majority of these models define the cost or utility function as a summation of quadratic costs including terms such as effort and accuracy, or even variance (e.g. [5]), because quadratic cost functions ensure tractable solutions [3]. However, recent studies have proposed relatively simple cost functions that more accurately predict the behavioral decisions of people than quadratic cost functions (e.g., [6,7]). Inclusion of these cost functions should improve the predictive and explanatory power of our computational control models, provided these simple models can accurately generalize across various tasks of a similar nature.

Cost functions describe the relative merits of different choices. Selection of an appropriate cost function is important for a variety of reasons. For example, the majority of recently proposed cost functions predict compensatory behavior in the presence of skewed noise distributions, whereas a quadratic cost function does not. Implications like this one can have at least three effects. First, they lead to more accurate predictions of user’s behavior. Second, they cause us to become cognisant of phenomenon and questions of which we might otherwise be unaware—for example, is the biological noise distribution skewed? In our own field of myoelectric control, for example, we did not think to raise the question until the language of cost functions made it relevant. Once we did pose the question, we realized that due to the rectification of the stochastic signal, processed myoelectric signals are highly skewed. Finally, having accurate cost functions enables more accurate causal assignment of measured behavior, in contrast to incorrectly attributing a phenomenon that is in fact due to the cost function to some other portion of the model such as adaptation or estimation. Thus cost functions form an important pillar in the field of computational motor control, in which we seek cost functions that are simple, accurate, and generalize across parameter values within a given category of tasks. Recent studies have provided relatively simple cost functions that improve accuracy, but the question remains: do they generalize?

**Fig 1:**
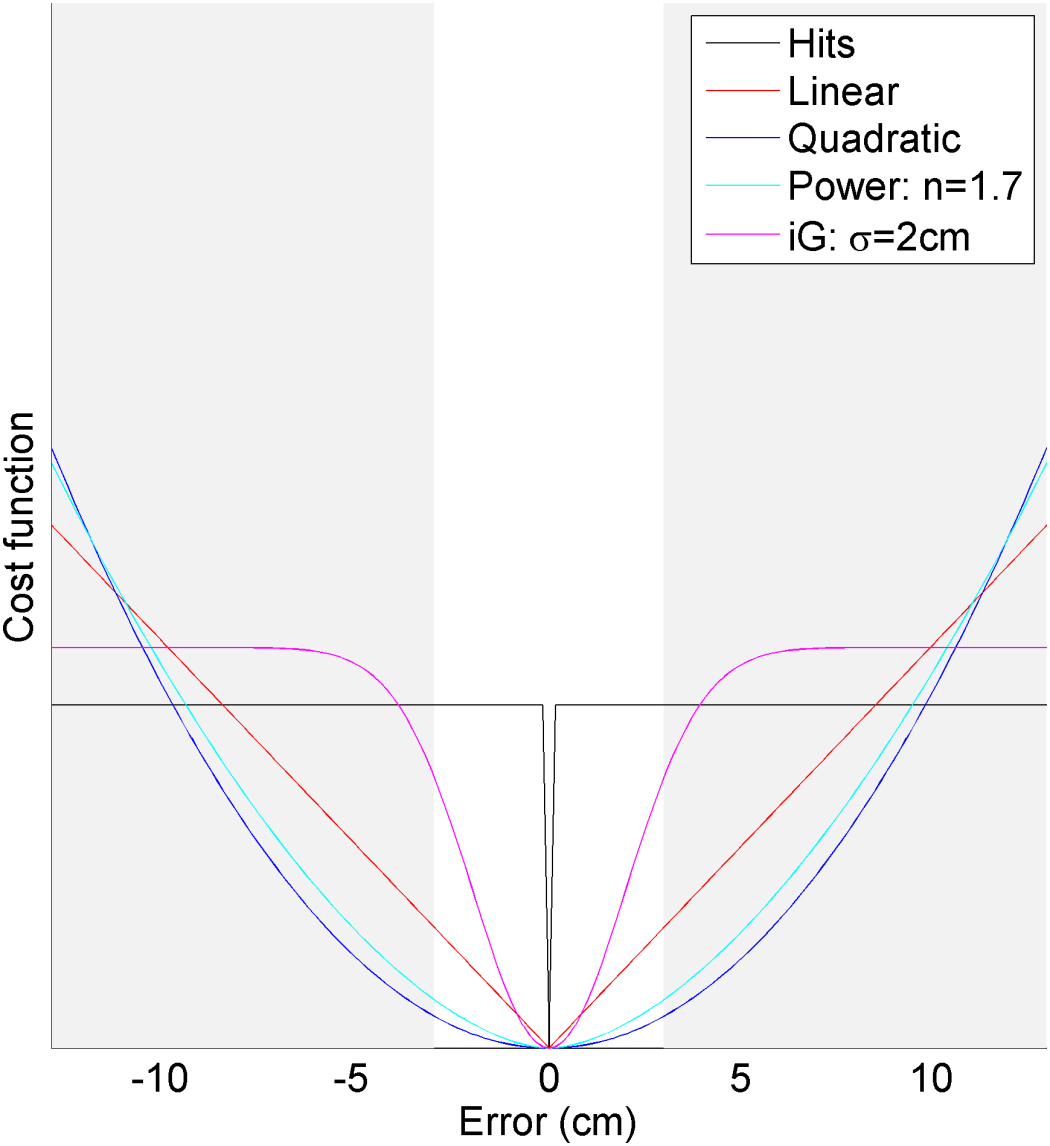
Examples of possible cost functions, including a Hits model (all non-zero errors are punished equally), linear and quadratic functions, and two parametric functions: a power model and an inverted Gaussian (iG) model. Power and iG parametric models are shown with the values Kording obtained across subjects controlling an interface with low inherent noise. The x-axis range is indicative of the maximum error experienced in this experiment, which used noisier control signals. The white region is the range of error considered by Kording’s experiment. If subjects use the same cost function for noisier signals, there should be a notable difference between the iG and power models for high levels of error: the iG model in particular should flatten out. If, however, people scale the model found in Kording’s study relative to the range of error encountered, the cost function would look like a scaled version of the white range of the figure.

Cost functions can exist as a trade-off between two different quantities, such as time vs. effort [8] or variance vs. effort [9]; or it can be a trade-off between the probability of two different values of the same quantity, such as small error vs. large error (e.g., [6,7]). This study focuses on cost functions across a single quantity (in this case, error), because such cost functions seem more likely to generalize across scale. A quadratic cost function, for example, penalizes large errors disproportionally to small errors, whereas a linear function penalizes large errors proportionally to small errors. Kording and Wolpert [6] have shown that for systems with minimal inherent noise, such as pointing your finger, the cost function is well represented by an inverted Gaussian cost function or by a power cost function (e.g., ψ*∼*|*error*|^1.7^). Taking the example of the inverted Gaussian cost function, for large errors, the cost function is essentially flat (Fig 1)—the user cares about all errors equally, regardless of magnitude. This cost function poses an interesting example to address the question of generalization: for systems that have *large* inherent error, such as myoelectric control of prostheses [10], do the parameters acquired in small-error studies generalize (e.g., flat cost function for large errors)? The purpose of this study was to answer this question on a typical human-machine interface that inherently involved large noise and a dynamic task.

This paper used the inverse-decision-theory technique of Kording and Wolpert [6] to reconstruct a cost function from empirical data. Subjects were asked to track a moving target using myoelectric control signals, which have substantial inherent noise, and their resulting signal was biased by a skewed distribution. Subjects accordingly shifted their response, based on both the level of skewness and their own inherent cost function. Unlike Gaussian noise, where shifting by the mean is always the optimal solution, the optimal shift for a skewed distribution depends on the particular cost function employed, allowing us to distinguish between candidate cost functions. By calculating what cost function results in this same relationship between skewness and shift, we estimated the cost function used by the subjects. The nature of this cost function for signals with large multiplicative noise, compared with previously inferred cost functions, is an indication of how consistent user’s cost functions are across varying tasks.

## Materials and Methods

### Overview of the method

**Fig 2:**
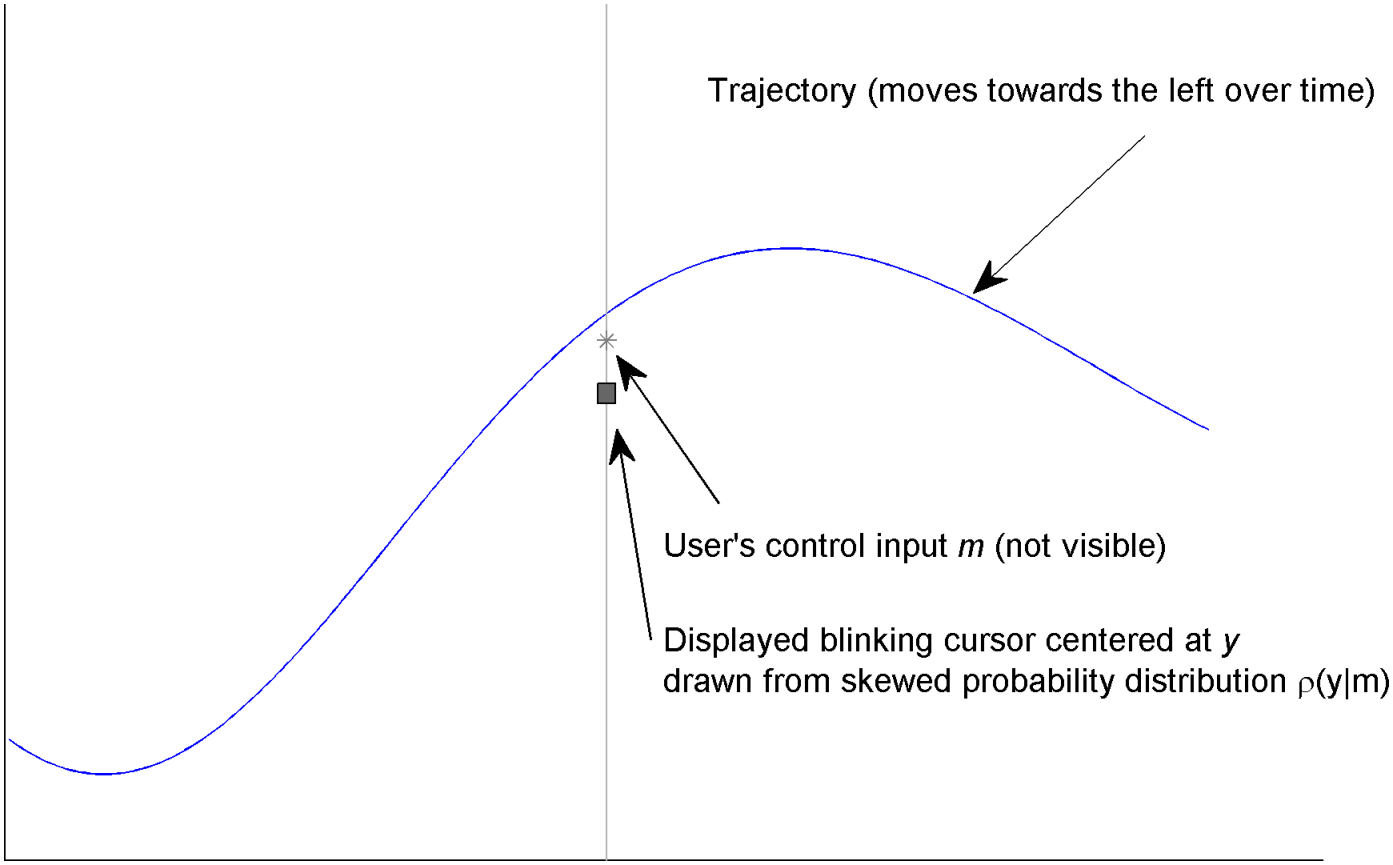
Visual Display. A trajectory moves across the screen from right to left. The subject moves the cursor up and down in the middle of the screen. The displayed cursor blinks at 1 Hz, and is drawn from a skewed distribution that is shifted by the user’s control input *m*. The subject is asked to, on average, keep the cursor on the waveform.

**Fig 3:**
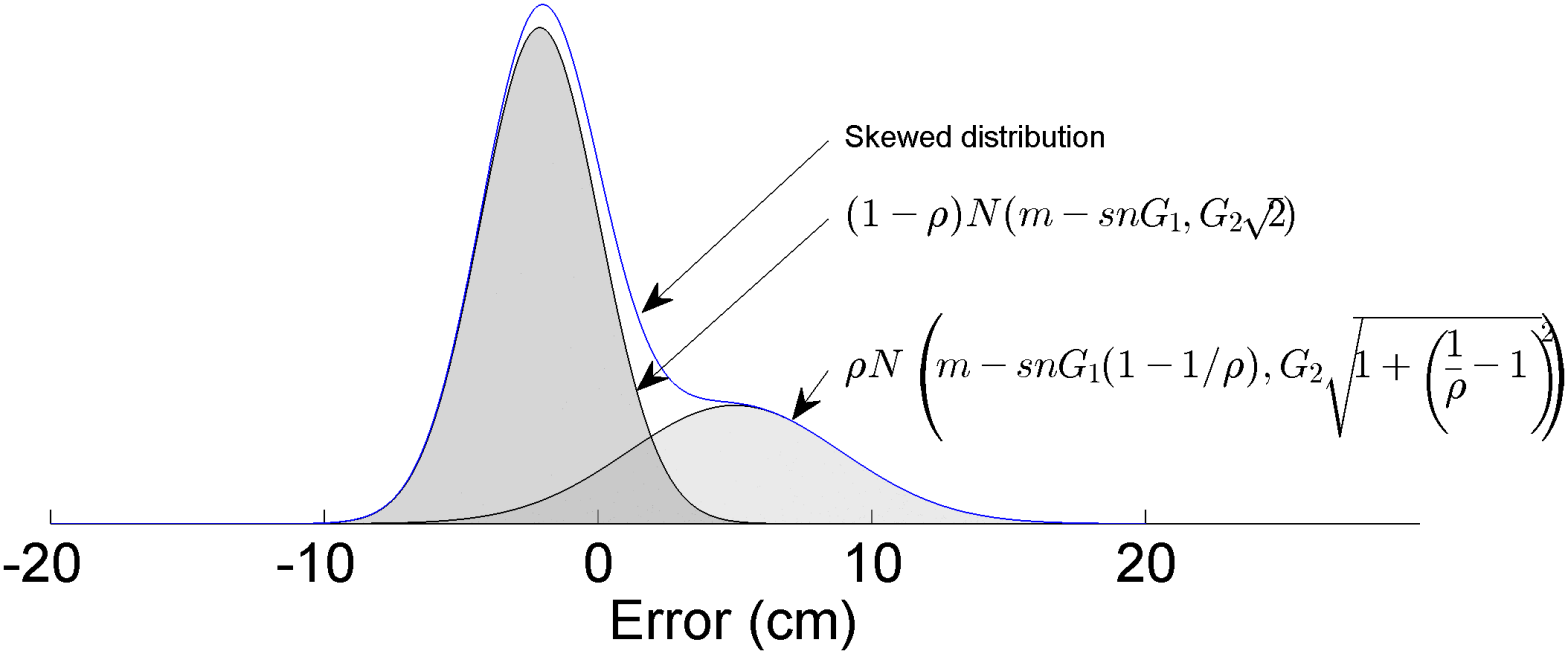
Skewed probability distribution was generated by sampling from two Gaussian distributions with different means. Given the nature of the equation, the skewed distribution had the same mean (*m*), regardless of skewness ρ.

Users watched a random signal progress across the screen from right to left in this tracking task. They controlled a cursor in the middle of the screen that was able to move up and down, using myoelectric signals to affect its velocity (Fig 2). The vertical position *y* of the blinking cursor was drawn from a skewed probability (Fig 3):

$$p\left(y|m\right)=\;\left(1-\rho \right)N\left(m-snG_{1},G_{2}\sqrt{2}\right)+\rho N(m-snG_{1}\left(1-1/\rho \right),G_{2}\sqrt{1+{\left(\frac{1}{\rho }-1\right)}^{2}}),$$ (1)

Where *N*(*μ*, *σ*) is a Gaussian centered at *μ* with standard deviation *σ*, *m* is the control signal of the user, *ρ* is the level of skewness (ranging from no skewness (*ρ* = 1) to infinite skewness (*ρ* = 0), *sn* is a randomized sign variable to avoid a bias, and *G*_*1*_ and *G*_*2*_ are gain variables that tune the size of the distribution. *G*_*2*_ affects the standard deviation of the skewed distribution, and must be set high enough relative to the user’s inherent noise to be noticeable. *G*_*1*_ affects the shift between the distributions. The level of skewness *ρ* was fixed for a given trial and the median shift *m* of the user across the trial was recorded.

Users could have a variety of cost functions, ranging from one in which all non-zero errors are punished equally, to a linear cost function, to a quadratic cost function. Kording and Wolpert found that a power function and an inverted Gaussian relationship both described user’s cost functions well for signals with small inherent error. Each of these distributions is shown in Fig 1. Because the probability distribution used in this study is shifted by the user’s response *m*, the user can affect the total cost by adjusting *m: cost=∫ψ(y)p(y|m)dy*. For a given cost function *ψ* and a skewed probability distribution, a certain value of *m* results in the minimum cost. We can accordingly compare a user’s shift *m* as a function of skewness ρ, to the optimal shift *m* for a variety of candidate cost functions ψ.

### Experimental protocol

**Fig 4:**
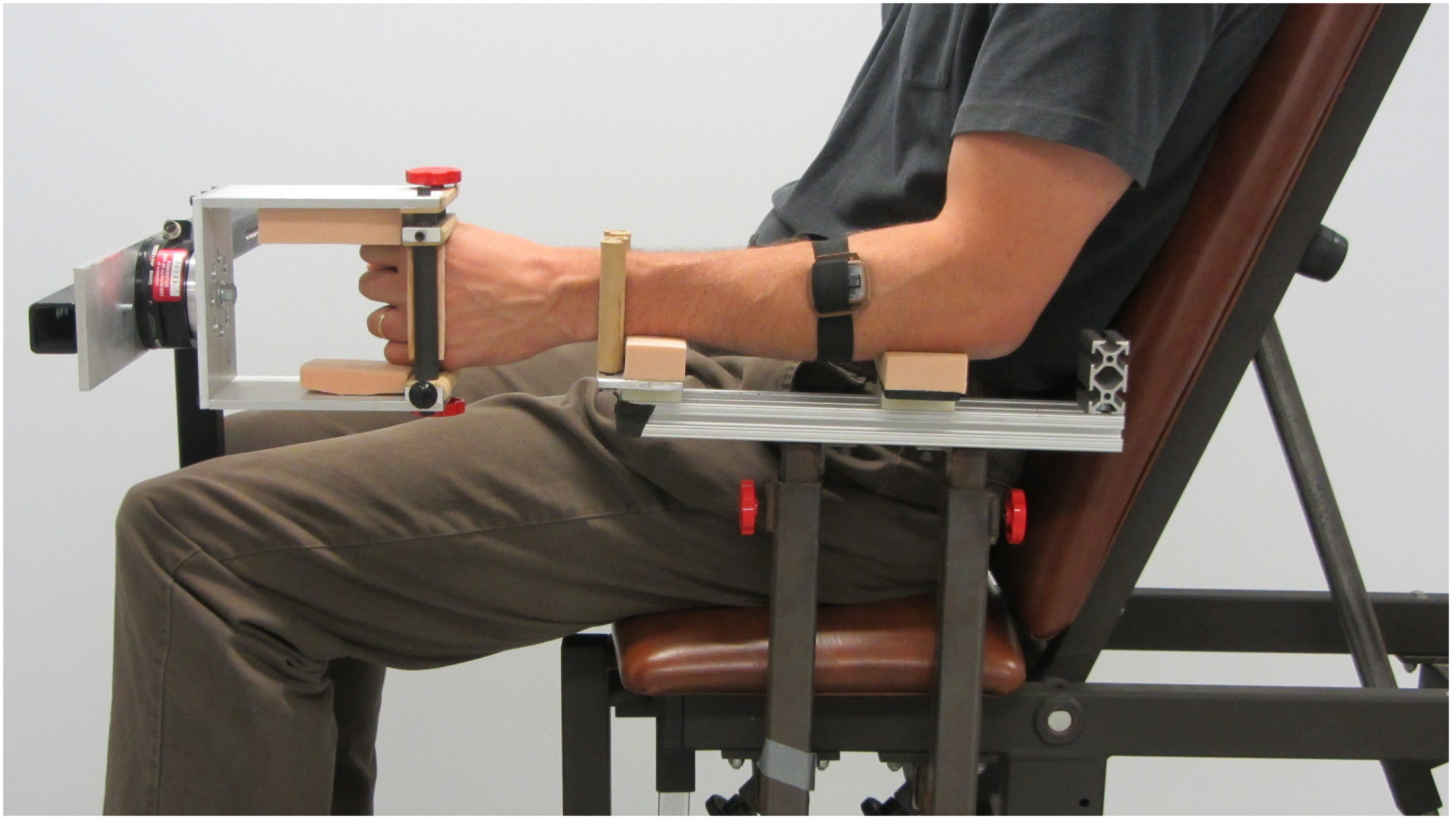
Experimental setup. Surface electrodes were cuffed on the forearm. The hand grasped a fixed rod, and the forearm was held in place by two adjustable padded rods.

After providing written informed consent, six female and 18 male naïve subjects (aged 19–44) participated in this study. The experiment was carried out in accordance with institutional guidelines. The University of New Brunswick—Fredericton campus’s Research Ethics Board approved the experimental protocol. The individual photographed in Fig 4 of this manuscript has given written informed consent (as outlined in PLOS consent form) to publish these case details.

Subjects sat in a custom chair 1.5m away from a 713x1211 mm large-screen monitor and gripped a post with their dominant arm. Padded rods were adjusted on either side of their forearm to prevent movement of the wrist (Fig 4). Two wireless Delsys Trigno electrodes in an elastic cuff were placed over their forearm extensor and flexor muscle groups. Thresholds and gains of the two signals were calibrated in a custom GUI [11] until subjects could easily and independently generate myoelectric control signals. The difference between the calibrated signals was mapped to the vertical velocity of the screen’s cursor, which blinked on and off at 1 Hz.

Subjects were asked to track a waveform moving from right to left across the screen on average as close to the target as possible. The waveform was composed of the summation of six sinusoidal signals with frequencies of [.05 .06 .07 .08 .09 .1] Hz, with amplitudes inversely proportional to the frequencies [12]. The phase of each sine-wave was randomly chosen for each trial from a uniform distribution, creating a pseudorandom trajectory that moved across the screen with a maximum frequency of 0.1 Hz, enabling it to be easily tracked. This low-frequency content, coupled with the ability of subjects to preview the upcoming signal before it reached their cursor, ensured minimal phase-delay between the commanded position and subject’s control of the cursor. Subjects performed twelve blocks of 30 trials each. Each trial lasted 12 seconds, and the first two seconds were discarded. Subjects were given a 2-second rest between trials and a 1-minute rest between blocks.

Subjects saw the actual position of their control input on the first and last block. On the remaining 10 blocks, the distribution of their cursor was skewed using Eq (1), where the level of skewness uniformly varied each trial between 0.1 and 0.8. The values of G_1_ = 2.135cm and G_2_ = 1.54cm were determined during a pilot study of four subjects and were chosen such that the standard deviation of the noise added by the distribution was approximately 1.5X as large as the inherent noise in tracking the waveform without artificial noise.

### Candidate Cost functions

The median shift in each user’s control signal in response to the skewed distribution was binned across values of ρ = 0.1 to = 0.8 in 0.05 increments. For a given candidate cost function and value of *ρ*, the optimal shift in the user’s signal to minimize the total cost (cost=∫ψ(y)p(y|m)dx) was calculated using Matlab’s *fminsearch* function. The parameters of the candidate cost function were in turn tuned using Matlab’s *fminbnd* function to minimize the sum of squared error (SSE) between the empirical shift and the optimized shift of the candidate cost function. Distributions were assessed across three standard-deviations of error (e.g. -62–62 cm) in order to ensure that the code returned optimal shifts for a given probability distribution function and cost function.

Six candidate cost functions were considered that were monotonic and symmetric [6,7]. The first (termed *hits*) weighted all non-zero errors equally, for which the optimal shift is to align the mode with the target. We also considered a quadratic function, for which the optimal shift is to align the mean (which in our skewed probability distribution was equal to the user’s shift *m*, regardless of the skewness *ρ*). We considered a linear function, for which the optimal shift can be analytically shown to be the median of the distribution, which for the case of our distribution lies between the mode and the mean. Finally, we considered three parametric cost functions. Power and inverted Gaussian cost functions each had one tunable parameter, whereas a recently proposed quadratic cost function with a non-zero linear term [7] has two tunable parameters (quadratic and linear gains).

The total sum of squared error (SSE) of each candidate cost function was assessed per subject. Both SSE and parameter values across subjects tended to have skewed distributions (Jarque-Bera p-values <0.05), typically with a right-handed tail. Nonparametric statistics were accordingly used. Friedman’s test, a nonparametric version of a balanced two-way ANOVA, was used to compare values across subjects, using a post-hoc analysis with Tukey Kramer correction to look for differences between potential cost functions using Matlab’s function *multcompare*. Median and interquartile parameter values were reported.

## Results

**Fig 5:**
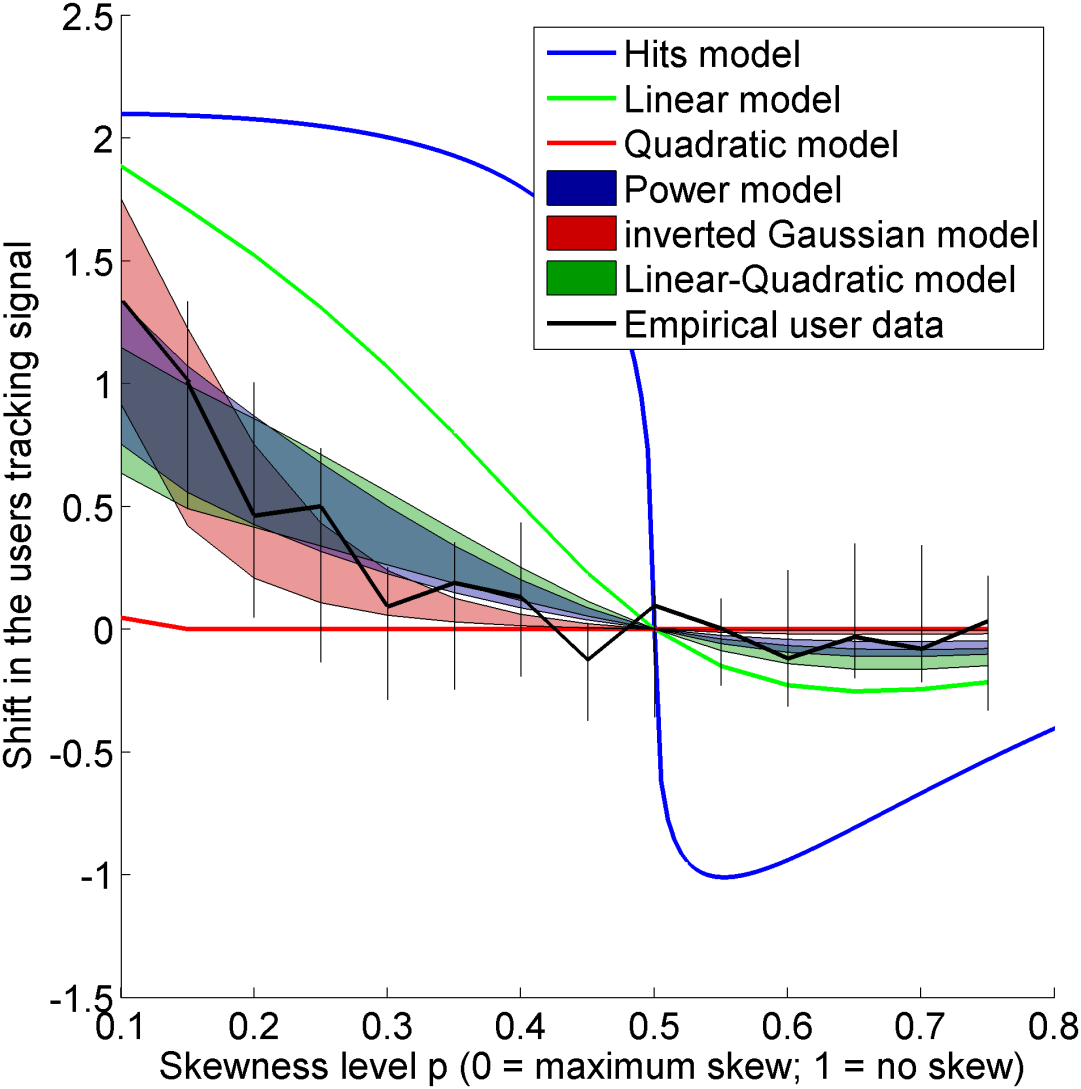
Empirical shifts caused by a skewed noise distribution (median values shown, with inter-quartile vertical bars). Optimal shifts according to candidate cost functions are also illustrated, including the interquartile range of the three parametric models.

**Fig 6:**
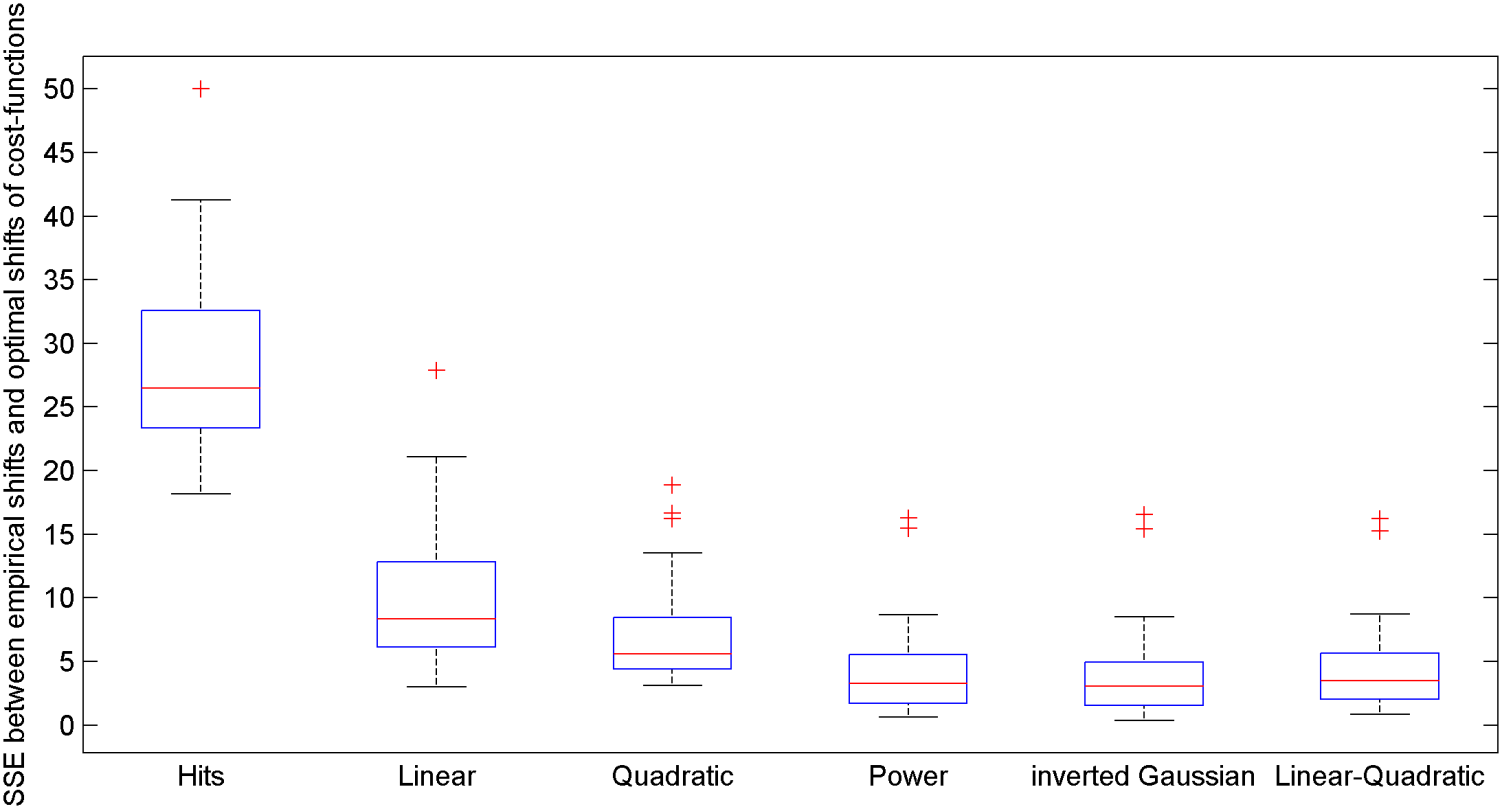
Accuracy of the candidate cost functions in predicting the empirical shifts.

The goal of this study was to evaluate candidate cost functions by comparing their optimal shifts *m* against the empirical shifts *m*, across a range of skewed distributions varied by parameter *ρ*. Fig 5 shows the median shift of 24 able-bodied subjects as a function of skewness. It also shows the shift predicted by various cost functions. In general, their response fell between the distributions of a linear and a quadratic function. Power, inverted Gaussian, and linear-quadratic cost functions were fit to these data, and the optimal shifts of those functions, shown across the interquartile range of their tuned parameter, are shown as well. It can be seen that each parametric cost functions represented the response of users across the range of skewness tested. It can also be seen that the shift in the user tracking signal corresponded well to the amplitude predicted by the optimal models. All three of the parametric models were able to capture the behavior of the subjects and were statistically different from the linear and quadratic models, but there was no significant difference across subjects between the three parametric models. SSE values across the models are shown in Fig 6.

**Fig 7:**
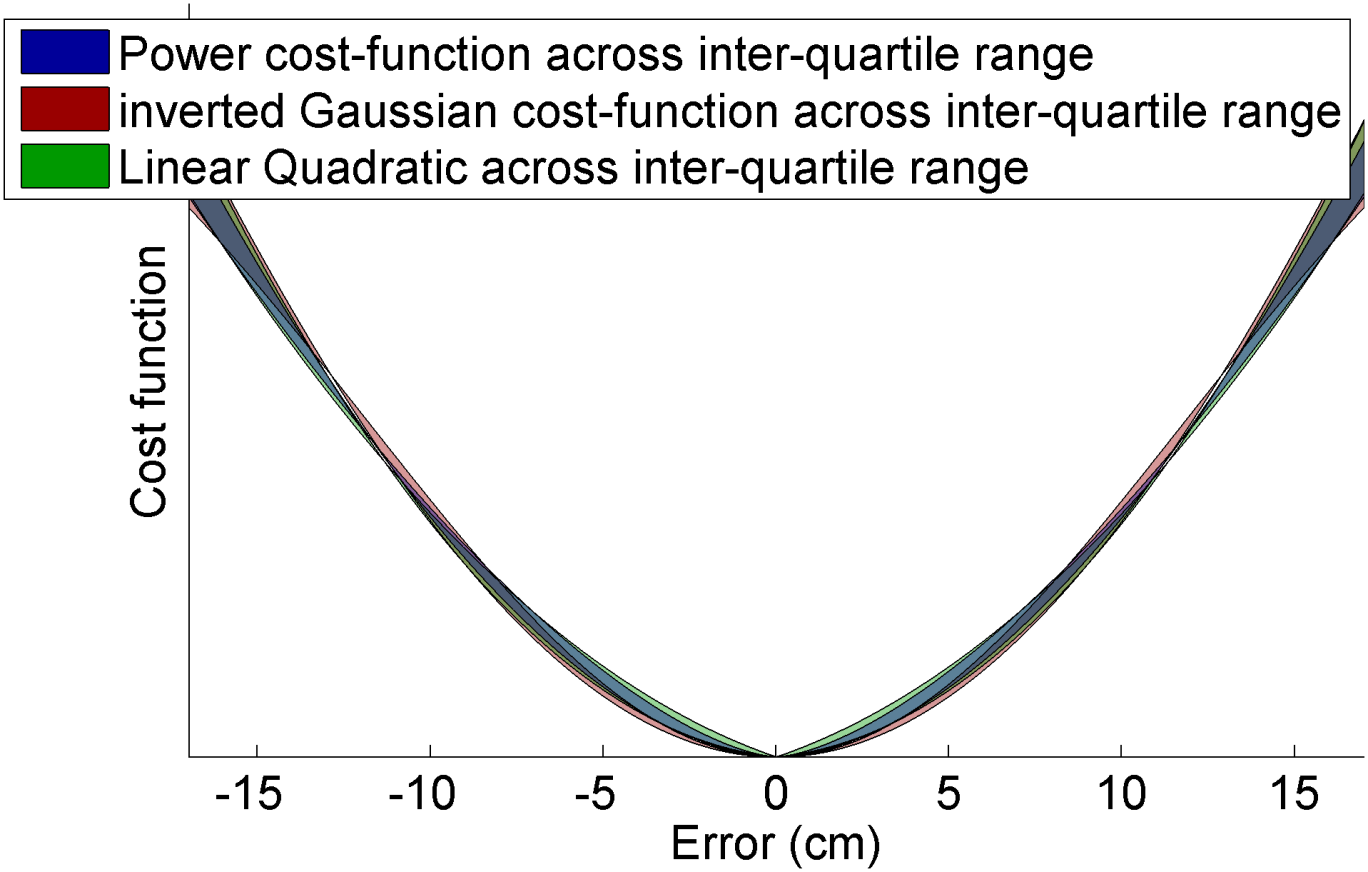
Interquartile range of parametric candidate cost functions across the range of error encountered in this study. Note that the inverted Gaussian distribution does not flatten out for the optimum parameter set and over the range of error encountered.

The main goal of this study was to see if users’ cost functions generalized to larger errors. The parameter value for the power model (*ψ∼*|*error*|^*n*^) was *n* = 1.69, with an interquartile range of [1.53–1.78]. Note that this value is indistinguishable from Kording’s fit of 1.67 ± 0.23 standard deviation. The tuned parameter value for the inverted Gaussian ($\psi \sim -e^{-\;\frac{error^{2}}{2\sigma^{2}}}$) was σ = 17.3 cm, with an interquartile range of [11.8–26.1] cm. Compared with Kording’s fit of σ = 2.03 ± 0.23 cm standard deviation, the spread is significantly larger, and the resulting fit, shown in Fig 7, looks like a scaled version of Kording’s function (shown in the white region of Fig 1), rather than a generalization of it (shown in the full width of Fig 1). It appears that subjects do not generalize a given inverted-Gaussian cost function across larger errors for tracking tasks. Thus, we may infer two things for tracking tasks: first, that cost functions do not flatten out for large errors, but rather scale a given cost function across that error. Secondly, the power-relationship is accordingly more ubiquitous in describing this relationship than the inverted Gaussian model, since it is a dimensionless parameter that does not depend on the spread of the error.

The linear-quadratic cost function fit a cost function of *ψ* = *a*_1_ *error* + *a*_2_ *error* ^2^. The empirical range of parameters were a_1_ = 0.00755 [.00753 .00756] and a_2_ = 0.0017 [.0008 .0045]. Niu et al. [7] found that a_1_ varied across subjects, but that a_2_ was consistently 1.78±.24 N. However, it is difficult to compare Niu’s parameters to the ones obtained in this study, since Niu et al.’s task was to grasp an object with five fingers (force manipulation), whereas the tasks used by Kording [6] and ourselves were both motion tasks. The characteristics of a linear-quadratic (in which the coefficients are blended) are similar to the characteristics of a power function (in which the power falls between 1 and 2). We accordingly compared the accuracy of the power-function directly to the linear-quadratic, and found that although the linear-quadratic had more tunable parameters, it resulted in a statistically worse fit of empirical shift across subjects (p<0.01). The power-function accordingly seems to best represent the cost function for tracking tasks in terms of generalization and accuracy.

It should be noted that after a brief familiarization session, subjects found the task easy to perform, given the intuitive nature of the control interface and the slow tracking frequency. No skewed noise was added during the first and last block, to serve as baselines. Subjects had median absolute error of 1.13cm and 0.75 cm during these blocks: two orders of magnitude noisier than the control signal used in Kording’s study (0.025cm).

## Discussion

In order to be useful for predicting how users perform in a category of tasks, a candidate cost function should be both accurate and generalizable across the parameters of that category. This study used an inverse-decision-theory technique to compare the response of users with the response of candidate cost functions across a range of skewed distributions. The evaluation technique was the same as Kording and Wolpert’s technique [6]. The task itself fell in the same category of motion accuracy, but involved tracking of a dynamic waveform rather than pointing at a static target; used a velocity interface between the user’s control signals and the monitor rather than a position interface; and involved two orders of magnitude more inherent noise in the user’s control signal. The task accordingly served as a good platform to assess the accuracy and generalization of candidate cost functions from Kording and Wolpert’s study to the broader class of motion tracking.

Our study found that a variety of parametric cost functions were accurate, but that only one—a power-function—generalized across the studies. It is worth noting that although an inverted Gaussian model flattens off for large errors (e.g., *err* ≫ *σ*), once the parameter had been fit to subject data the model did not flatten out within the region of errors tested in Kording’s study (e.g., white region of Fig 1, err_max_ = 3cm, σ_tuned_ = 2cm) or this study (Fig 7 err_max_ = 17cm, σ_tuned_ = 11.8–26.1cm). Thus, although Kording and Wolpert proposed two candidate cost functions for motion tasks (inverted Gaussian and power), the results of both their study and this appear to narrow it down to a single cost function of a power function as the best model to capture the cost function of subjects for tracking tasks.

A power cost function is a useful generalization that can be applied to models of human control of tracking and pointing tasks that currently use a quadratic cost function. The power-function parameter found in both studies (1.67–1.69) is sufficiently close to a quadratic function that for most applications, algorithms can be optimized using a quadratic cost function, which has known convergence properties. However, once the settings have been optimized, they should be rerun with a cost of 1.68 rather than 2. It is likely that they will be sufficiently close to the global maximum to converge (e.g., [13]).

There are several implications of a non-quadratic cost function. The most immediate is the case in which the user interacts with a system that has skewed noise, in which the optimal response will depend on whether their cost function is quadratic or to the 1.68th power. Although the majority of studies typically assume a Gaussian noise distribution, skewed distributions are fairly prevalent. For example, to use the example of this study—myoelectric control—even if we assume the heteroscedastic noise in the raw EMG signal is Gaussian [10], as soon as we rectify the signal in preparation to estimate the envelope [14] the noise distribution has a substantial positive skew. Additionally, estimation of features from non-stationary data EMG (e.g. during force-varying contractions) results in significant skew [15]. To go a step farther, within the realm of pattern recognition [16], many features (time-domain, autoregressive) exhibit skewed distributions. These examples serve to illustrate that for many human-machine interfaces, we should potentially reevaluate whether the noise has a skewed distribution in light of the fact that it will affect user’s behavior if they use a power cost function, as well as the corollary: that we should evaluate whether modeling user’s with a 1.68^th^ power provides more accurate estimates of their behavior.

Even for interfaces with Gaussian noise, learning rates will be affected depending on whether the user has a quadratic or a 1.68^th^ power cost function—it would adapt less in each trial. Trial-by-trial adaptation has often been studied within computational motor control; it would be interesting to apply a non-quadratic cost function to the topic to assess the impact on tracking tasks.

This study used an inverse-decision-theory to assess candidate cost functions by adding skewed noise to user’s signals. There are at least three limitations of this approach that should be noted. First, it is possible that the introduction of added noise influenced people’s behavior. In other areas of computational motor control, studies have shown that people respond differently in response to self-generated error than to added error [17,18]. Taken to an extreme, the use of added noise can cause people to completely distrust the visual display or to give up trying to do their best job to remain accurate. To a lesser extent, it seems likely that people will integrate their own perception of the error they expect to see with the visual feedback that they receive, and that the behavioral compensation we measure is accordingly a filtered version that incorporates not only the skew introduced in the visual feedback, but also their own internal understanding of what the noise properties should be.

Second, because we are use an inverse decision theory, our conclusion is only as good as our assumption that a cost function should result in a shift in the user’s average signal: any method that only indirectly measures the interested phenomenon is only as strong as its inferences. For example, it is possible that people have a different cost function, but also do not have an optimal controller, and that if we indirectly construct the cost function by assuming an optimal controller, we end up recreating a different cost function than the user actually has. In our case, people do appear to behave optimally even in the presence of large noise sources [19], although if subjects have a misconception of the prior, this can result in suboptimal control [4]. As another example, our assumptions do not incorporate risk sensitivity in that they only look at the average shift across a trial, and it has recently been shown that in the face of increased uncertainty (such as imposed by our added noise), subjects become more risk adverse [5]. In any case, our conclusions must be softened by our dependency on our assumptions, both explicit and implicit.

Finally, we only evaluated six candidate cost functions, and it is possible that the way people behave in tasks such as the one we tested is actually closer to an untested cost function. Kording and Wolpert addressed this by fitting a non-parametric cost function [6], but in our work we found the non-parametric fitting algorithm to be too sensitive to parameter choices for which we had no good rationalization—in our experience a substantially larger number of data points with smaller bin windows would be required to be confident in the estimate provided by a non-parametric cost function. Even non-parametric cost functions typically have some constraints, such as symmetry or smoothness, and recent studies have suggested that at least some biological cost functions such as muscle-effort, the cost function is not smooth [20]. Thus we may only conclude that of the candidate cost functions we evaluated, a power-law is the most descriptive.

Despite these limitations, a power cost function does seem like a useful candidate cost function. Like any good scientific tool, it is both simple and accurate: it generalizes across orders of magnitude of error; it generalizes across dynamics; and most importantly, it provides a reasonable estimate of user’s behavior, thus enabling it to be integrated into the larger field of computational motor control.
